# Morphological Biomarker Differentiating MCI Converters from Nonconverters: Longitudinal Evidence Based on Hemispheric Asymmetry

**DOI:** 10.1155/2018/3954101

**Published:** 2018-03-19

**Authors:** Xiaojing Long, Chunxiang Jiang, Lijuan Zhang

**Affiliations:** Paul C. Lauterbur Research Center for Biomedical Imaging, Shenzhen Institutes of Advanced Technology, Chinese Academy of Sciences, 1068 Xueyuan Avenue, Shenzhen 518055, China

## Abstract

Identifying subjects with mild cognitive impairment (MCI) who may probably progress to Alzheimer's disease (AD) is important for better understanding the disease mechanisms and facilitating early treatments. In addition to the direct volumetric and thickness measurement based on high-resolution magnetic resonance imaging (MRI), hemispheric asymmetry could be a potential index to detect morphological variations in MCI patients with a high risk of conversion to AD. The present study collected a set of longitudinal MRI data from 53 MCI converters and nonconverters and investigated the asymmetry differences between groups. Asymmetry variation was observed in the medial temporal lobe, especially in the entorhinal cortex, between converters and nonconverters 3 years before the former developed AD. The proposed asymmetry analysis was observed to be sensitive to detect morphological changes between groups as compared to the methods of voxel-based morphometry (VBM) and thickness measurement. Hemispheric asymmetry in specific brain regions as a neuroimaging biomarker can provide helpful information for prediction of MCI conversion.

## 1. Introduction

Alzheimer's disease (AD) is the most common form of dementia with a rapidly rising prevalence worldwide as a result of global aging. There are currently no treatments available that can provide a cure for AD. However, major scientific advances in genetical, biochemical, cell biological, and neuroscience studies over the last decades offer hope that effective therapeutic intervention is likely to be developed and to largely change the life quality of AD patients if the disease is detected prior to the onset of overt symptoms and signs [1].

Mild cognitive impairment (MCI) manifests as an intermediate stage between the expected cognitive decline in normal aging and the more serious decline in age-associated dementia [2]. It causes a slight but measurable decline in cognitive abilities; nevertheless, it does not significantly impact patients' daily functioning. People with MCI may be three to five times more likely to develop AD or other types of dementia relative to those without MCI [3]. However, not all people with MCI get worse and some even get better. Identifying the subjects with MCI who will end up with AD (referred to as “MCI converters”) is of great importance in terms of better understanding the disease mechanisms and initiating early treatments for the patients.

Combined with the clinical tests of cognition or behaviors, biomarkers from structural and functional magnetic resonance imaging (MRI) have been employed for the disease characterization. Volumetric analysis for the whole brain, hippocampus, and ventricles has demonstrated different atrophy or enlargement rate in annual change between MCI converters and subjects with stable MCI with a larger tissue loss in MCI converters [4–8]. Voxel-based morphometry (VBM) and tensor-based morphometry (TBM), which allow 3D mapping of focal differences in brain anatomy, have detected significantly faster tissue reduction in the medial temporal lobe, posterior cingulate gyrus, and hippocampus [9–12]. Surface-based analysis which provides insight into the cortical morphological variations has identified shape differences between MCI converters and nonconverters in the inferior parietal cortex and both lateral and medial temporal cortices, especially in the entorhinal cortex [11, 13, 14].

Previous studies have also demonstrated asymmetrical cerebral atrophy in AD and MCI. Barnes et al. [15] and Shi et al. [16] have, respectively, reported asymmetric atrophy in the hippocampus. Derflinger et al. have found that lobar asymmetry of the temporal, parietal, and occipital lobes is different from healthy controls to AD patients [17]. In our earlier study, significant asymmetry alterations in limbic structures were identified among healthy elderly, MCI converters, and AD patients [18], suggesting that structural asymmetry difference may embed biomarkers that distinguish MCI converters from nonconverters.

In this study, a multivariate model that collectively takes into account the effects of multiple morphological variables, including the cortical thickness, surface area, and curvature index, was used to investigate the hemispheric asymmetry on a longitudinal database to distinguish MCI converters from nonconverters. Images from 5 visits (1 baseline and 4 follow-ups) were examined to determine the earliest time point at which converters can be differentiated from nonconverters. The results were compared to those of the VBM and vertex-based thickness analyses for a sensitivity evaluation among different methods in discriminating patients of a high risk.

## 2. Materials and Methods

### 2.1. Subjects and MR Scanning

Data of 26 MCI converters (15 males, aged 73.35 ± 6.71 years) and 27 nonconverters (21 males, aged 73.01 ± 7.47 years) was retrieved from the Alzheimer's Disease Neuroimaging Initiative (ADNI) database (http://adni.loni.usc.edu/), phases 1, Go, and 2. ADNI is the result of efforts of many investigators from a broad range of academic institutions and private corporations, and subjects have been recruited from over 50 sites across the US and Canada. The ADNI study was approved by the IRB of all participating sites. All subjects and, if applicable, their legal representatives gave written informed consent prior to the collection of clinical, genetic, and imaging data. The dataset follows the rigor establishment with careful quality control, detailed documentation, and full anonymization prior to data analysis. Approval for public sharing of the anonymized data was obtained.

The subjects are right handed and had taken at least five follow-up visits with MR scanning at an interval of roughly one year. The MCI nonconverters were not diagnosed with probable AD in any of the visits. The MCI converters remained in the MCI category for at least 3 consecutive visits before conversion to AD and maintained mild AD status for at least 2 consecutive visits after conversion.

High-resolution T1-weighted images (T1WI) were obtained using the MP-RAGE sequence with typical parameters of TR = 6.5~9.5 ms (GE)/2300~3000 ms (SIEMENS), TE = 3.0~4.0 ms, FA = 8°, FOV = 256 × 256, and in-plane resolution = 1 mm × 1 mm × 1.2 mm. The ADNI MRI core has established strict guidelines and procedures to ensure similar image qualities across sites and platforms over time. A customized MP-RAGE pulse sequence was developed for the GE platform to minimize vendor-to-vendor differences [19].

### 2.2. Hemispheric Asymmetry Analysis

Data were processed with the “recon-all” pipeline of FreeSurfer software (Version 5.0.0, http://surfer.nmr.mgh.harvard.edu/). Images were first intensity normalized, skull stripped, aligned to a standard space, and segmented into gray matter (GM), white matter (WM), and cerebrospinal fluid (CSF). The cortical surface and WM surface were constructed, inflated, topologically corrected, and projected to the standard spherical coordinate system defined by the FreeSurfer brain atlas. Then, the cerebral cortex was automatically divided into 34 gyral-based regions of interest (ROI) [20–22]. The surface area, mean curvature index, and cortical thickness were calculated on each ROI of each hemisphere. ROI-wise multivariate analysis of covariance (MANCOVA) was performed to test the compositive morphological differences between the left and right hemispheres for each group. For each ROI, the surface area, curvature index, and cortical thickness were introduced as collective response variables with gender and age as covariates. Regions with significant asymmetry were identified as *P* < 0.05. Correction was performed in multiple comparison procedures to control the false discovery rate (FDR) at 0.05. *P* values were mapped onto the cortical surface of the left hemisphere of FreeSurfer *fsaverage* template for visualization.

The asymmetry index (AI) of each ROI for each subject was defined as

(1) $$\begin{array}{c} {AI}^{j}=\sum_{i}\frac{L_{i}^{j}-R_{i}^{j}}{L_{i}^{j}+R_{i}^{j}}, \end{array}$$

and the group asymmetry index of each ROI was defined as

(2) $$\begin{array}{c} AI={avg}_{j}\left(\sum_{i}\frac{L_{i}^{j}-R_{i}^{j}}{L_{i}^{j}+R_{i}^{j}}\right), \end{array}$$

where *L*_*i*_^*j*^ denotes the *i*th variable of the left hemisphere for the *j*th subject and *R*_*i*_^*j*^ denotes the *i*th variable of the right hemisphere for the *j*th subject, avg_*j*_(*x*) denotes an average of *x* across the group. AI > 0 indicates leftward lateralization, and AI < 0 indicates rightward lateralization. Between-group difference for a specific region was computed using the two-sample *t*-test on the asymmetry index AI^*j*^ with gender and age effects being regressed out. The significance level was set at 0.05.

### 2.3. Voxel-Based Morphometry (VBM)

Cross-sectional VBM analysis was performed for each time point. SPM8 was used for voxel-based morphometry (http://www.fil.ion.ucl.ac.uk/spm/). Images from all groups were first roughly aligned via a rigid body transformation and segmented into gray matter, white matter, and CSF [23]. The template image used for spatial normalization was created by averaging across the study cohort. The segmented gray matter tissue map was aligned from the individual space to the template space, scaled by the Jacobian field, and smoothed with the full width at the half maximum (FWHM) of 10 mm, followed by a modulation step to correct for volume change [24]. A design matrix was constructed, and the general linear model (GLM) was fitted to the data with gender and age as covariates. Voxel-wise parametric statistical tests were performed to compare the GM differences between MCI converters and nonconverters. Corrections for multiple comparisons were conducted using the theory of Gaussian random fields (GRF) as the preprocessed images were spatially smoothed and voxels were correlated with their neighbors. Significant voxels with corrected *P* < 0.05 were displayed on the MNI brain template for visualization.

### 2.4. Vertex-Based Thickness Analysis

Cross-sectional thickness analysis was performed for each time point. FreeSurfer software (Version 5.0.0, http://surfer.nmr.mgh.harvard.edu/) was used for image processing and group analysis. Processing steps were conducted as introduced in hemispheric asymmetry analysis. Surface-based normalization was computed to map thickness data of each subject onto a common group space that allows the thickness to be compared across subjects at homologous points on the cortex. The thickness is then smoothed with FWHM = 10 mm, and the GLM was fitted to the data with gender and age as covariates (*FreeSurfer*_*Qdec*). Vertex-wise statistical test was performed to compare the thickness differences between converters and nonconverters for each hemisphere. Corrections for multiple comparisons were conducted to control the FDR at 0.05. The corrected significance map with *P* < 0.05 was finally overlapped onto the *fsaverage* brain template surface for visualization.

## 3. Results

No significant difference was found in age and MMSE scores at the baseline visit between converters and nonconverters (Table 1).

### 3.1. Hemispheric Asymmetry Analysis

**Figure 1:**
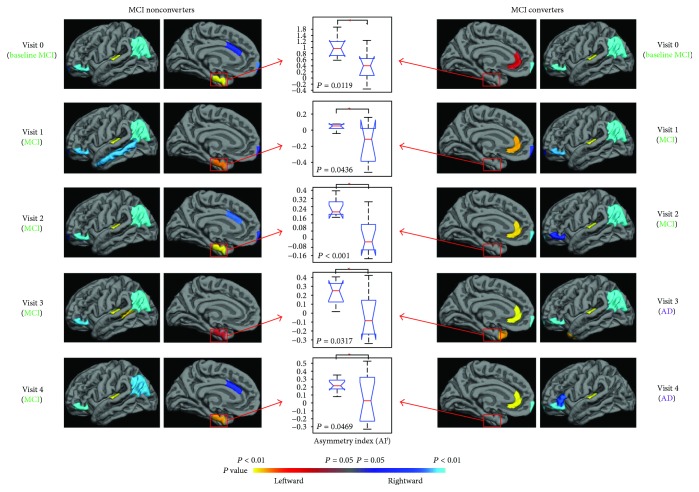
Hemispheric asymmetry analysis demonstrated different patterns in the medial side of the brain between the MCI nonconverters and converters at all time points. The MCI nonconverters showed leftward lateralization in the entorhinal cortex, while the converters showed rightward lateralization in the rostral cingulate gyrus. Significant between-group difference of the asymmetry index AI^*j*^ was observed in these two regions with *t*-test analysis (middle column).

Both MCI converters and nonconverters featured leftward lateralization in the transverse temporal gyrus, as well as rightward lateralization in the lateral orbitofrontal gyrus and inferior parietal lobule (Figure 1). Different asymmetry patterns were identified in the medial side of the brain, specifically located in the limbic system including the anterior cingulate cortex and the entorhinal cortex. Leftward lateralization was observed in the entorhinal cortex in MCI nonconverters from baseline to the last visit, as well as in the rostral anterior cingulate gyrus in converters during all visits. Rightward lateralization in the caudal anterior cingulate gyrus was detected at the baseline and the 2nd and 4th follow-up visits in MCI nonconverters.

**Figure 2:**
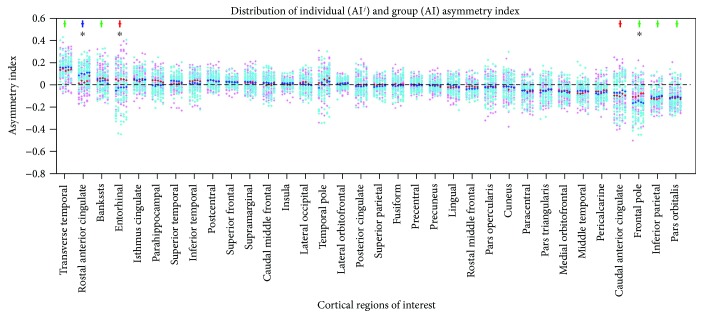
Distribution of individual and group asymmetry indices for 34 cortical regions. For each region, a column represented indices of one visit. Pink and red dots, respectively, represented individual and intragroup mean asymmetry indices of MCI nonconverters. Cyan and blue dots, respectively, represented individual and intragroup mean asymmetry indices of converters. Colored crosses denoted that significant asymmetry was observed in the corresponding region in either or both groups, where red crosses denoted significant asymmetry in nonconverters, blue denoted asymmetry in converters, and green denoted asymmetry in both groups. Black asterisks denoted that significant between-group difference was observed in the corresponding region. Bankssts: banks of the superior temporal sulcus.

**Figure 3:**
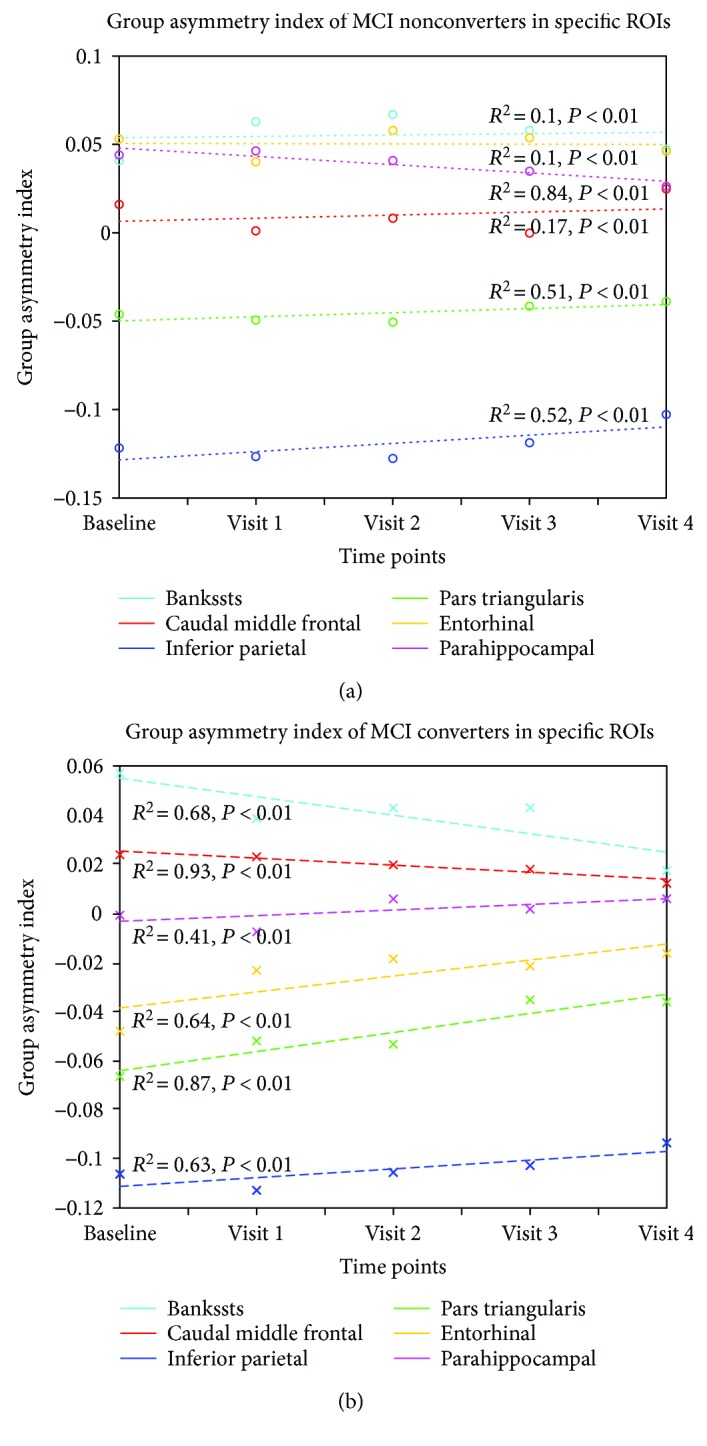
Regression of group asymmetry indices for MCI nonconverters and converters in five selected ROIs, including the banks of the superior temporal sulcus (cyan), caudal middle frontal gyrus (red), inferior parietal lobule (blue), pars triangularis (green), entorhinal cortex (yellow), and parahippocampal gyrus (pink). Group asymmetry indices decreased more notably to 0 in MCI converters from baseline to follow-up visits compared to nonconverters. Bankssts: banks of the superior temporal sulcus.

Calculation of the group asymmetry index of each ROI at all visits showed that a certain of the 34 cortical regions that underwent asymmetry decreases with the mean asymmetry index trending towards zero over time (Figure 2). Those regions included the language-related areas such as the banks of the superior temporal sulcus, caudal middle frontal gyrus, inferior parietal lobule, and pars triangularis, as well as the memory-related areas such as the entorhinal cortex and parahippocampal gyrus (Figure 3). MCI converters exhibited a more notable decreasing trend than nonconverters, especially in areas of the banks of the superior temporal sulcus, the entorhinal cortex, and the pars triangularis. In some other regions, including the posterior cingulate, superior parietal lobule, fusiform, precentral gyrus, and precuneus, group asymmetry indices varied slightly over 5 visits and the values approximately equal zero (Figure 2). Two-sample *t*-test analysis demonstrated that significant between-group differences were found in the entorhinal cortex, frontal pole, and rostral anterior cingulate. No significant between-group difference was observed in other ROIs.

### 3.2. Voxel-Based Morphometry

**Figure 4:**
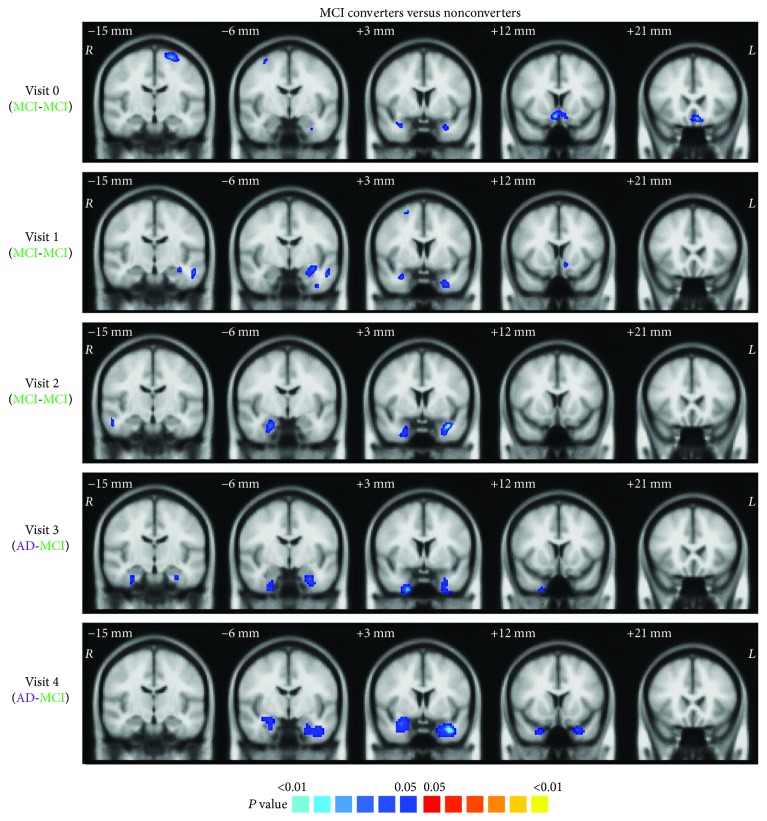
Voxel-based morphometry demonstrated notable and consistent volume reduction located in the hippocampus, amygdala, entorhinal cortex, and parahippocampal gyrus in the MCI converters from the baseline to the last visit.

The MCI converters were observed with greater tissue shrinkage from the baseline scanning to follow-ups in the bilateral hippocampus, amygdala, parahippocampal gyrus, and entorhinal cortex, compared with nonconverters (Figure 4). Significant volume reduction in converters was also observed in the precentral gyrus, superior frontal gyrus, middle temporal gyrus, and nucleus accumbens at the first two visits.

### 3.3. Vertex-Based Thickness Analysis

**Figure 5:**
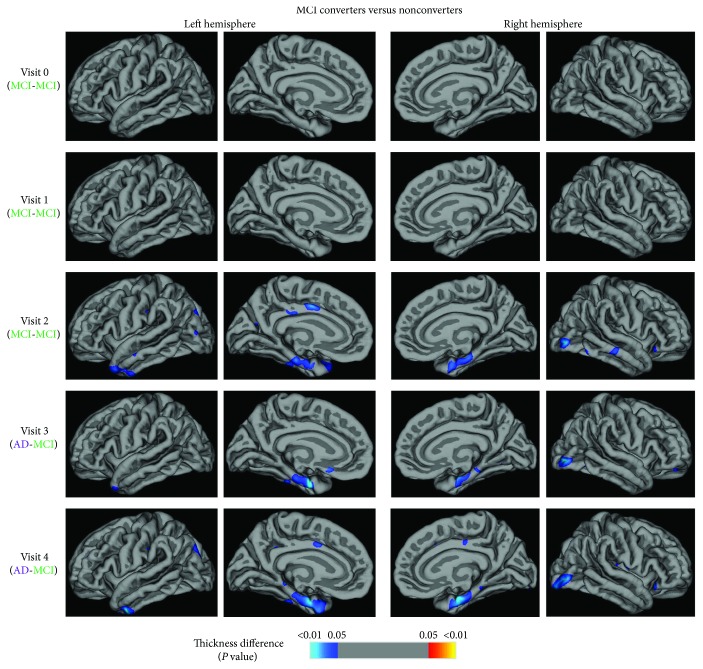
Vertex-based thickness analysis demonstrated bilateral thinner cortices in the lateral occipital gyrus, entorhinal cortex, temporal pole, and cingulate cortex in MCI converters from 1 year before they progressed to AD, where the entorhinal cortex presented the most severe shrinkage.

No significant thickness difference was found between the MCI converters and nonconverters in the first two visits. From 1 year before the converters progressed to AD, we observed thinner cortices mainly located in the bilateral occipital and temporal lobes, specifically in the lateral occipital gyrus, entorhinal cortex, temporal pole, and cingulate gyrus (Figure 5).

## 4. Discussion

In this study, cross-sectional hemispheric asymmetry analysis as well as VBM and vertex-based thickness analyses was performed for different time points to identify regions that could discriminate MCI converters from nonconverters and how early they could work for differentiation. The results demonstrated that asymmetry and VBM analyses were sensitive to morphological differences between the two cohorts, which can be used to dissociate MCI converters 3 years prior to conversion.

VBM and vertex-based thickness analyses have been commonly used to capture the dynamics of gray matter atrophy and cortical thickness reduction in AD and MCI patients [12, 25, 26]. Chételat et al. have found significantly greater gray matter loss in MCI converters relative to nonconverters 18 months prior to their conversion in a longitudinal VBM study [12]. Risacher et al. have reported that MCI converters demonstrated higher atrophy progression with more severe reduction in gray matter density from baseline to 1 year [25]. They have also shown that annual thickness changes in MCI converters were significantly greater than those in nonconverters. In this work, we applied both VBM and thickness analyses along with the proposed asymmetry method on the same set of longitudinal data to detect the morphological differences between MCI converters and nonconverters. The VBM study identified remarkable volume reduction in MCI converters 3 years before they progressed to AD, whereas vertex-based thickness analysis only detected significant cortical thinning in converters 1 year before conversion. Hutton et al. showed that thickness analysis provided a more sensitive measure of age-associated decline in gray matter compared with VBM [27]. However, the present study demonstrated that VBM works better in differentiating slight variation between age-matched brains, which may be attributable to the difference in methodology that thickness analysis selectively investigates cortical thickness while VBM provides a mixed measure of gray matter including cortical surface area or cortical folding, as well as cortical thickness [27]. The proposed asymmetry method has also shown great power of sensitively detecting group differences. In our previous study, we have found that multivariate analysis that combined multiple morphological measures including the surface area, curvature index, and cortical thickness was more sensitive in picking up subtle alterations of hemispheric asymmetries than each individual measure [18]. None of the individual measures have acted as the pivotal contributor [28], but the compositive effect of all measures enhanced the performance. Studies have illustrated that gray matter atrophy is asymmetric in AD and MCI [17, 18]. The present work showed a consistent result but further demonstrated that the asymmetric tissue shrinkage occurred at least 3 years earlier to the conversion of AD. The asymmetry alteration could be a novel and sensitive imaging marker that can distinguish MCI patients with a high risk of conversion to AD at an early time point.

Brain asymmetry is believed to be evolutionally adaptive, enabling more specialized and efficient functioning of the brain [29]. Gómez-Robles et al. have suggested that morphological asymmetry of the human brain enhances cognition compared to that of nonhuman primates [30]. Plessen et al. have assessed the correlation of cognitive performance with measures of cortical thickness asymmetry in children and adults and reported that asymmetry in cortical thickness is correlated with human cognitive function [31]. In this study, regression analysis of group asymmetry indices demonstrated a more pronounced decreasing trend from baseline to follow-up visits in MCI converters, compared with nonconverters, in the language-related and memory-related cortical regions, including the banks of the superior temporal sulcus, caudal middle frontal gyrus, inferior parietal lobule, pars triangularis, entorhinal cortex, and parahippocampal gyrus. The observation is consistent with the common understanding that AD is characterized by progressively worsening deficits in language [32] and memory [33]. Among the aforementioned cortical regions, the asymmetry indices of the entorhinal cortex, banks of the superior temporal sulcus, and pars triangularis demonstrated the sharpest decline in converters yet gentle variations in nonconverters, which agreed with our previous finding that hemispheric asymmetry was better preserved in these regions in healthy elderly and in the early stage of MCI converters but lost in AD patients [18]. Killiany et al. have reported that MRI measures of the entorhinal cortex and the banks of the superior temporal sulcus were most useful in predicting the follow-up status of MCI subjects [34]. These evidences suggested that the discriminative characteristics of the featured regions may consist not only in the volume reduction of corresponding structures but also in the imbalance of tissue atrophy. Although converters and nonconverters were both diagnosed with MCI with a degree of cognitive decline at the baseline visit, the brain morphological patterns of the two groups have differed at an earlier time point.

Asymmetry indices in the posterior cingulate gyrus, superior parietal lobule, fusiform, precentral gyrus, and precuneus smoothly ranged around zero in both MCI converters and nonconverters, indicating very mild asymmetry from baseline to follow-ups. Nevertheless, the results may arise from different situations. Regions such as the fusiform, precentral gyrus, and precuneus presented no significant asymmetry in young adults and healthy elderly [18, 35], suggesting that the absence of asymmetry may intrinsically exist and may not be affected by aging or cognition decline. However, pronounced asymmetry was observed in regions such as the posterior cingulate and superior parietal lobule in young adult brain [18, 35] while it disappeared in healthy elderly and MCI subjects, which may imply that asymmetry in these regions varies over time but is not distinctive to differentiate between healthy elderly, MCI converters, and nonconverters.

Converging evidence has demonstrated that gray matter shrinkage in prodromal AD mainly occurred in the medial temporal lobe (MTL), specifically in the hippocampus, parahippocampal gyrus, and entorhinal cortex. By measuring the hippocampal and entorhinal volume, deToledo-Morrell et al. have shown that the volumes of these two regions can well differentiate MCI converters from nonconverters, especially the right hemisphere entorhinal cortex that best predicted conversion with an accuracy of 93.5% [36]. Risacher et al. have reported that the degree of degeneration of MTL tissue was the best antecedent MRI marker of conversion, where hippocampal volume decreases were the most robust [11]. Another study has found that cortical thickness was significantly reduced in MCI within the MTL network [37]. The results showed in the present study that the MTL structures, especially the entorhinal cortex, are the best predictor to identify MCI patients with a high risk for AD which was consistent with previous findings [38]. As a system specifically important for declarative memory [39], the MTL is the first brain area to succumb to neurofibrillary tau pathology in AD [40]. Significant morphological changes in MTL, which were detected in MCI converters 3 years before they progressed to AD, could be an effective biomarker for early diagnosis or prediction of AD.

In conclusion, hemispheric asymmetry analysis identified the entorhinal cortex as a significant morphological biomarker to differentiate MCI converters from nonconverters. Asymmetry variation demonstrated the ability to identify morphological changes in MCI converters 3 years prior to conversion. Focal hemispheric asymmetry decreases progressively from an early time point of prodromal AD, which provides helpful information that facilitates the prediction of MCI conversion and the patient management in the clinical practice.

## Figures and Tables

**Table 1:** Baseline demographic information of MCI converters and nonconverters.

Groups	Number of subjects (m/f)	Age	APOE A1	APOE A2	MMSE score
Converters	26 (15/11)	73.35 ± 6.71	3.19 ± 0.49	3.73 ± 0.45	27.19 ± 2.05
Nonconverters	27 (21/6)	73.01 ± 7.47	2.93 ± 0.47	3.41 ± 0.5	28.07 ± 1.33

## Supplementary Materials

Supplementary Materials The IDs of subjects used in the study were listed in the supplementary file.
