# Supplementary material for: Morphological Biomarker Differentiating MCI Converters from Nonconverters: Longitudinal Evidence Based on Hemispheric Asymmetry
Source: Behav Neurol. 2018 Mar 19;2018:3954101. doi: 10.1155/2018/3954101 (PMC5884406; doi:10.1155/2018/3954101)
Supplement: Supplementary Materials — The IDs of subjects used in the study were listed in the supplementary file. [file 3954101.f1.doc]

**Supplementary File**

**Subject IDs**

| **MCI non-converters** | **MCI converters** |
| --- | --- |
| 002_S_1155 | 003_S_1057 |
| 002_S_1268 | 006_S_1130 |
| 003_S_0908 | 007_S_0041 |
| 003_S_1122 | 009_S_1030 |
| 005_S_0546 | 016_S_0702 |
| 014_S_0169 | 016_S_1117 |
| 018_S_0142 | 022_S_1351 |
| 021_S_0626 | 023_S_0042 |
| 022_S_1097 | 023_S_0126 |
| 027_S_0116 | 023_S_0217 |
| 027_S_0307 | 023_S_0331 |
| 029_S_0914 | 023_S_0376 |
| 029_S_1318 | 023_S_0388 |
| 031_S_0867 | 027_S_0408 |
| 032_S_0214 | 027_S_0835 |
| 036_S_0673 | 033_S_0922 |
| 041_S_0679 | 035_S_0204 |
| 041_S_1425 | 052_S_1346 |
| 053_S_0919 | 098_S_0160 |
| 057_S_1269 | 116_S_0361 |
| 127_S_0112 | 116_S_1243 |
| 127_S_0925 | 123_S_0108 |
| 127_S_1419 | 126_S_0708 |
| 128_S_0200 | 128_S_0227 |
| 129_S_1246 | 136_S_0695 |
| 130_S_0505 | 137_S_0994 |
| 136_S_0107 |  |
